# Associations among hedonic hunger, digital obesity, and type 2 diabetes self-management

**DOI:** 10.3389/fnut.2026.1825423

**Published:** 2026-05-21

**Authors:** Ayşe Çamli, Aylin Acikgoz Pinar, Merve Terzi

**Affiliations:** 1 Department of Nutrition and Dietetics, Graduate School of Health Sciences, Hacettepe University, Ankara, Türkiye; 2 Department of Nutrition and Dietetics, Faculty of Health Sciences, Hacettepe University, Ankara, Türkiye; 3 Department of Nutrition and Dietetics, Faculty of Health Sciences, Istanbul Yeni Yuzyil University, Istanbul, Türkiye

**Keywords:** diabetes self-management, digital lifestyle, digital obesity, eating behavior, hedonic hunger, type 2 diabetes mellitus

## Abstract

**Background:**

This study aimed to examine the mediating role of digital obesity in the association between hedonic hunger and type 2 diabetes self-management among adults with type 2 diabetes mellitus.

**Methods:**

This cross-sectional study was conducted in Turkey between April 28 and August 1, 2025. Data were collected using an online questionnaire, and 400 adults with type 2 diabetes mellitus were recruited through snowball sampling. The questionnaire included the Personal Information Form, the Power of Food Scale, the Digital Obesity Scale, and the Type 2 Diabetes Self-Management Scale. Multiple regression analysis was used to examine the associations of hedonic hunger and digital obesity with type 2 diabetes self-management. The mediating role of digital obesity was assessed using Hayes’ PROCESS macro (Model 4) with bootstrapping.

**Results:**

Hedonic hunger was positively associated with digital obesity and negatively associated with type 2 diabetes self-management. Digital obesity was also negatively associated with type 2 diabetes self-management. Mediation analysis indicated that digital obesity significantly mediated the association between hedonic hunger and type 2 diabetes self-management.

**Conclusion:**

Hedonic hunger was negatively associated with type 2 diabetes self-management, and digital obesity partially mediated this relationship. These findings highlight the potential importance of addressing both psychological eating tendencies and digital lifestyle behaviors in strategies aimed at improving diabetes self-management.

## Graphical abstract

**Figure:**
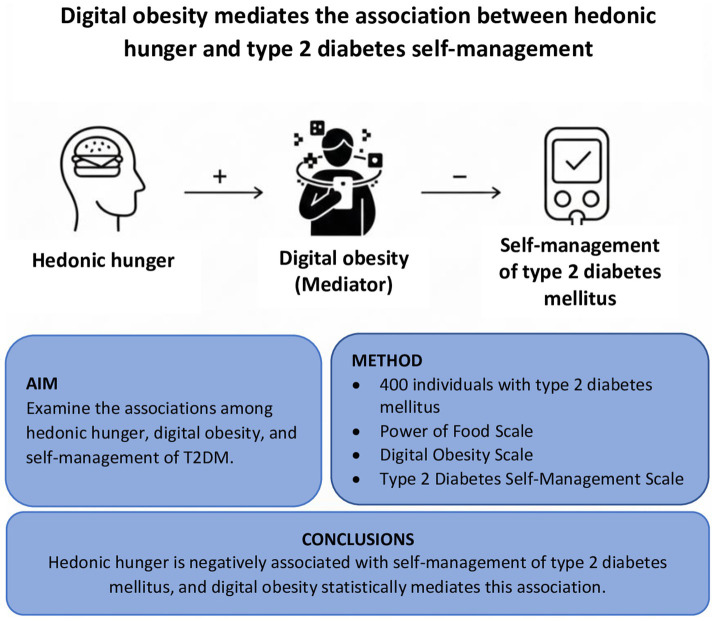

## 1 Introduction

Type 2 diabetes mellitus (T2DM) is a chronic metabolic disorder that has emerged as a major global public health concern, with a steadily increasing prevalence worldwide. According to the International Diabetes Federation (IDF) data for 2025, there are an estimated 589 million adults aged 20–79 with diabetes worldwide, and this number is projected to reach 853 million by 2050 (1). Effective management of T2DM relies heavily on sustained self-management behaviors, including adherence to a healthy diet, regular physical activity, appropriate medication use, maintenance of a healthy body weight, and regular monitoring of blood glucose levels (2). However, behavioral changes associated with modern lifestyles—particularly hedonic hunger and increasingly sedentary, screen-based behaviors—may pose growing challenges to optimal glycemic management among individuals with T2DM (3, 4).

Hedonic hunger refers to an individual’s propensity to eat for pleasure in response to emotional or environmental cues rather than physiological energy needs (5). This eating behavior has been linked to activation of the brain’s dopaminergic reward system and is further influenced by psychological factors such as stress, anxiety, and emotional dysregulation (6). Individuals with elevated levels of hedonic hunger tend to prefer highly palatable, energy-dense foods rich in sugar and fat, which may compromise adherence to dietary recommendations that are central to type 2 diabetes self-management (7). Higher consumption of such foods has been associated with increased body weight and a greater risk of obesity and T2DM (8, 9). Over time, hedonic eating behaviors have been linked to the concept of “diabesity”, reflecting the close coexistence of obesity and T2DM, both of which are associated with poorer glycemic control and difficulties in weight management—key components of effective type 2 diabetes self-management (10). Previous studies have reported associations between hedonic hunger and higher glycated hemoglobin (HbA1c) levels, poorer glycemic control, and increased obesity prevalence among individuals with T2DM (3, 11–15). Given that more than 89% of individuals with T2DM are overweight or obese and that weight reduction is associated with improvements in glycemic outcomes, understanding hedonic drivers of eating behavior has important clinical and public health implications (16–18). Beyond its association with glycaemic control and obesity, hedonic hunger may also be relevant to type 2 diabetes self-management through psychological and environmental pathways (19, 20). Previous research suggests that emotional eating, food cravings, psychosocial stressors, and difficulties in self-regulation may influence dietary behaviors and adherence-related outcomes in individuals with T2DM (21–28). Conceptually, hedonic hunger represents a non-homeostatic, reward-driven tendency that may interact with contextual influences such as food availability and social environment, thereby shaping self-management behaviors (3, 22, 26, 29, 30).

In parallel, the concept of digital obesity has recently emerged to describe lifestyle patterns shaped by excessive engagement with digital technologies. The term generally refers to behavioral patterns characterised by prolonged screen time, sedentary behavior, reduced physical activity, frequent exposure to digital food-related content, and increased reliance on online food environments (31–33). The widespread use of smartphones, social media platforms, and digital marketing has been associated with both decreased physical activity and increased consumption of energy-dense snacks (34). Extended screen exposure may not only reduce energy expenditure but also intensify hedonic eating tendencies through continuous exposure to food cues, potentially reinforcing dysregulated eating patterns (35). In the present study, digital obesity is operationalised as a behavioral pattern reflecting excessive screen exposure and digital engagement that may contribute to unhealthy lifestyle behaviors relevant to diabetes self-management. From this perspective, digital obesity may function as an important contextual factor in the association between hedonic hunger and type 2 diabetes self-management. Individuals with higher hedonic hunger may be particularly vulnerable to online food advertising, social media food content, and the convenience of digital food delivery services, which may be linked to less healthy dietary choices and reduced adherence to type 2 diabetes self-management behaviors (36). Conceptually, hedonic hunger reflects an individual-level motivational tendency driven by reward-related neural pathways that increases sensitivity to external food cues. In contrast, digital obesity primarily represents behavioral patterns associated with digital environments, including prolonged screen exposure, sedentary behavior, and increased interaction with online food-related content. Individuals with elevated hedonic hunger may therefore be more susceptible to these digital environments, which may reinforce reward-driven eating tendencies while simultaneously reducing opportunities for physical activity. Accordingly, digital obesity may represent a behavioral pathway through which hedonic hunger indirectly influences type 2 diabetes self-management behaviors. This proposed pathway is supported by previous research showing that greater digital media exposure is associated with unhealthy eating patterns, including higher snack and sugar-sweetened beverage consumption, as well as lower physical activity levels (37–40). In addition, studies on social media food content and digital food marketing suggest that online environments may intensify food cravings and reinforce preferences for highly palatable foods (41–47). Together, these findings support the view that digital environments may amplify reward-driven eating tendencies and undermine behaviors relevant to diabetes self-management. Previous studies have reported that higher levels of hedonic hunger are associated with poorer glycemic control, including higher glycated hemoglobin (HbA1c) levels, increased body weight, and reduced adherence to dietary recommendations among individuals with T2DM (3, 11–15). Furthermore, research has demonstrated that increased exposure to digital environments—such as prolonged screen time and online food-related content—is associated with higher consumption of energy-dense foods, more frequent snacking, and reduced physical activity (21–23, 45–48). These findings suggest that both hedonic hunger and digital lifestyle behaviors may adversely affect diabetes-related self-management. However, despite this growing body of evidence, the combined interplay between hedonic hunger, digital obesity, and type 2 diabetes self-management has not been adequately investigated. To the best of our knowledge, no study has specifically examined the potential mediating role of digital obesity in the association between hedonic hunger and self-management among individuals with T2DM. Therefore, the present study aimed to examine the associations among hedonic hunger, digital obesity, and type 2 diabetes self-management and to assess whether digital obesity mediates the relationship between hedonic hunger and diabetes self-management among adults with T2DM.

Hypotheses.

**Figure 1:**
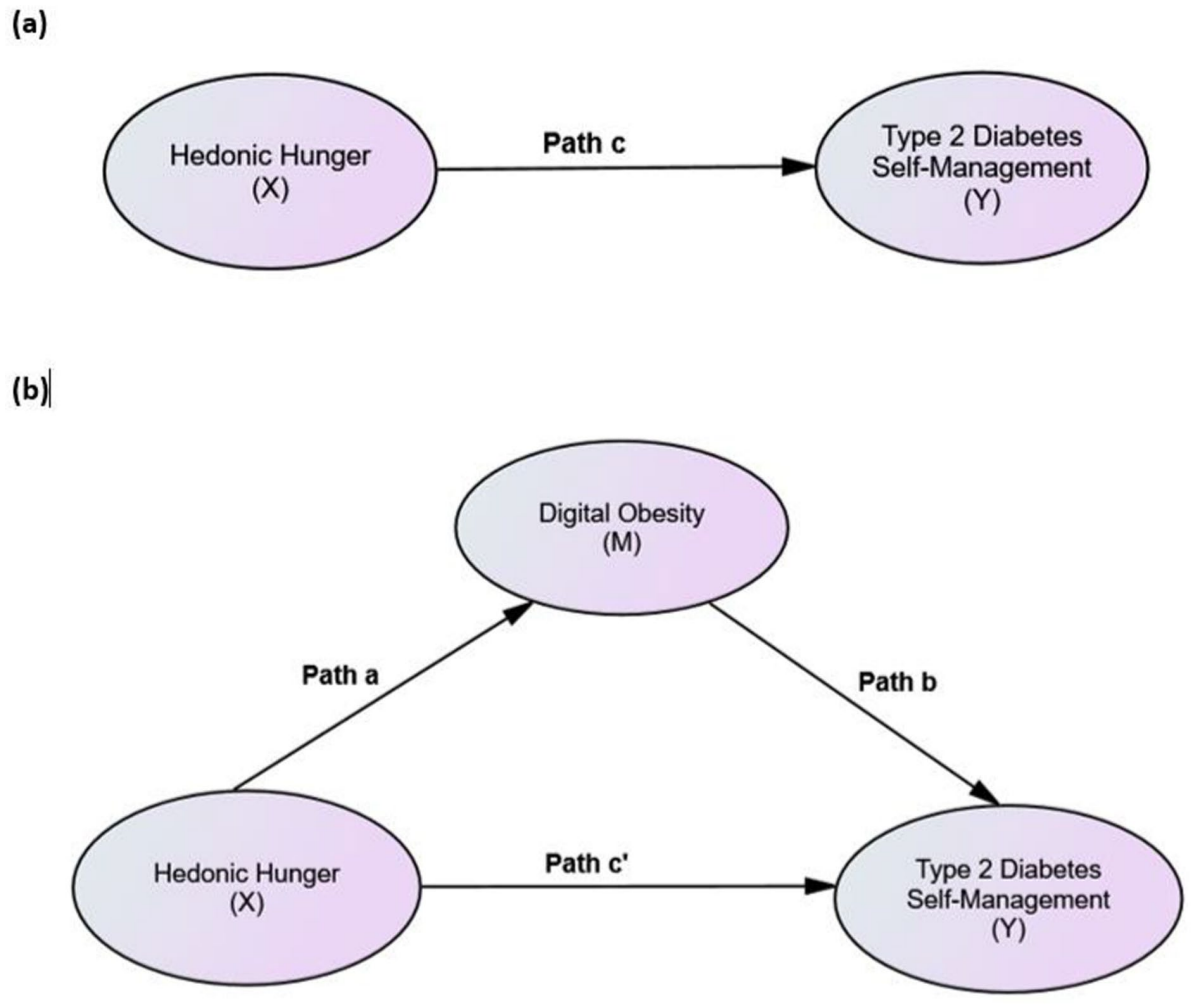
Conceptual mediation models examining the association between hedonic hunger and type 2 diabetes self-management: **(a)** total effect model (c path) and **(b)** mediation model including digital obesity, showing the direct effect (c′) and indirect effect (a × b).

The following hypotheses were examined using the proposed mediation model (Figures 1a,b):

*H1*: Hedonic hunger is significantly associated with type 2 diabetes self-management.

*H2*: Hedonic hunger is significantly associated with digital obesity.

*H3*: Digital obesity is significantly associated with type 2 diabetes self-management.

*H4*: Digital obesity mediates the association between hedonic hunger and type 2 diabetes self-management.

## 2 Methods

### 2.1 Study design and participants

This cross-sectional study was conducted among adults with T2DM between April 28, 2025, and August 1, 2025. The study population consisted of adults with T2DM residing in Turkey. Given the use of a non-probability sampling method, a formal sample size calculation based on probability sampling was not applicable. During the data collection process, responses meeting the predefined eligibility criteria were included in the analysis, resulting in a final sample of 400 participants. A *post hoc* power analysis was conducted using the G*Power 3.1 statistical software based on data from 400 participants, indicating a statistical power of 99% at a 95% confidence level with a medium effect size (48). Inclusion criteria were: (1) being between 18 and 64 years of age, (2) having been diagnosed with T2DM for at least one year, and (3) voluntarily agreeing to participate in the study by providing electronic informed consent. Exclusion criteria included: (1) being outside the 18–64 age range, (2) pregnancy or lactation, and (3) the presence of severe psychiatric disorders (e.g., schizophrenia, bipolar disorder, or other conditions that could impair the ability to complete the questionnaire reliably). Participants self-reported their diagnosis of T2DM in the questionnaire. Only participants who met the eligibility criteria were included in the final analysis. Ethical approval was obtained from the Erzurum Technical University Scientific Research and Publication Ethics Committee (Meeting No: 06, Decision No: 28, 2025) prior to the commencement of the study. Data were collected online using Google Forms. Participants were recruited using a non-probability snowball sampling method, in which the survey link was initially distributed through social media platforms (WhatsApp, Telegram, Instagram, and Facebook), and participants were encouraged to share the survey with other individuals diagnosed with T2DM. This approach facilitated the recruitment of participants from different regions of Turkey. All study procedures were conducted in accordance with data confidentiality principles, and participants were informed that the collected data would be used solely for academic research purposes. Electronic informed consent was obtained from all participants, who were required to approve the consent form before accessing the questionnaire. To minimise the risk of duplicate responses, the Google Forms survey was configured to allow only one response per participant, and the dataset was screened for potential duplicate entries prior to analysis.

### 2.2 Data collection tools

The study data were collected using the Personal Information Form, the Power of Food Scale (PFS), the Digital Obesity Scale and the Type 2 Diabetes Self-Management Scale. The Personal Information Form, prepared by the researchers, consisted of questions regarding gender, marital status, educational level, working status, smoking status, alcohol consumption, age, duration of diabetes, body weight, and height. Body mass index (BMI) was calculated as weight in kilograms divided by height in meters squared (kg/m^2^). BMI values were used for descriptive purposes and participant classification but were not included as covariates in the regression or mediation analyses.

**Figure 2:**
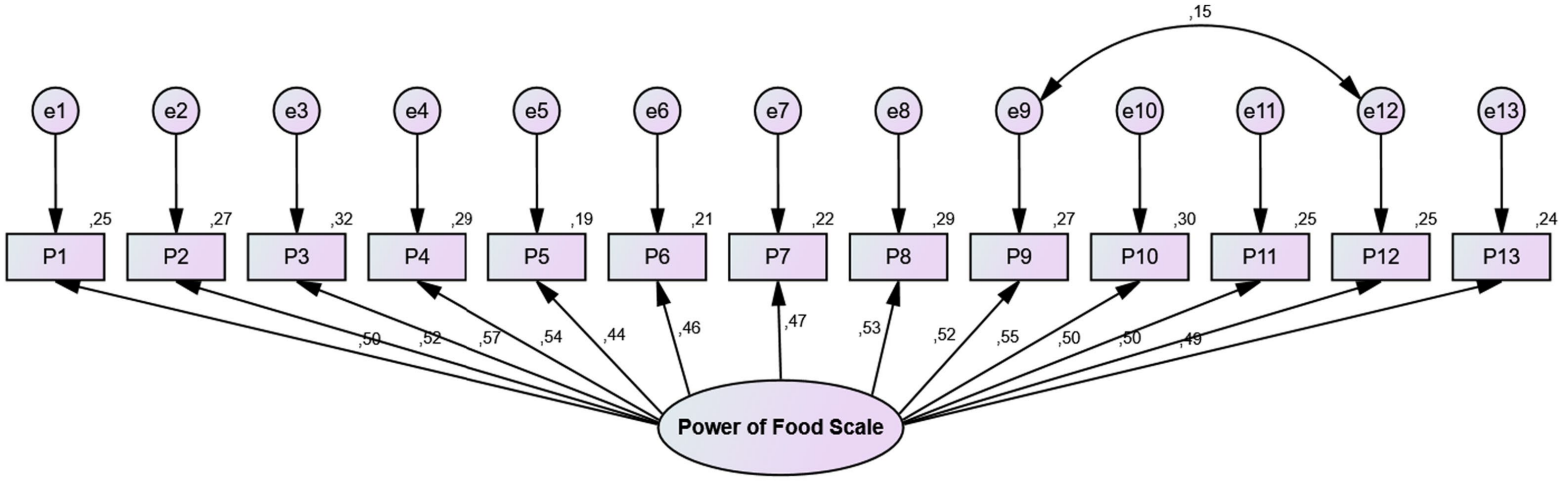
Confirmatory factor analysis (CFA) model for the Power of Food Scale (PFS) showing the factor structure and standardized factor loadings.

The Power of Food Scale (PFS) was developed by Lowe et al. (49) in 2009 to assess individuals’ psychological sensitivity to the environmental availability of food as an indicator of hedonic hunger. The scale was adapted into Turkish by Ülker et al. (50). The PFS consists of a total of 13 items and three sub-dimensions: the food available sub-dimension (items 1, 2, 9, and 10), the food present sub-dimension (items 3, 4, 5, and 6) and the food tasted sub-dimension (items 7, 8, 11, 12, and 13). The scale is a 5-point Likert-type scale, scored from 1 (strongly disagree) to 5 (strongly agree), and contains no reverse-coded items. Mean scores range from 1 to 5, with higher scores indicating greater levels of hedonic hunger. The Cronbach’s alpha coefficient of the original scale was reported as 0.92 (50). In the present study, the Cronbach’s alpha value was found to be 0.82. The construct validity of the Power of Food Scale was evaluated using confirmatory factor analysis (CFA). According to the results of the analysis, the fit indices were determined as χ^2^/df = 1.075, RMSEA = 0.014, CFI = 0.994, GFI = 0.975, AGFI = 0.964, IFI = 0.995, TLI = 0.993. The construct validity of the scale was supported by the CFA results (51) (Figure 2).

**Figure 3:**
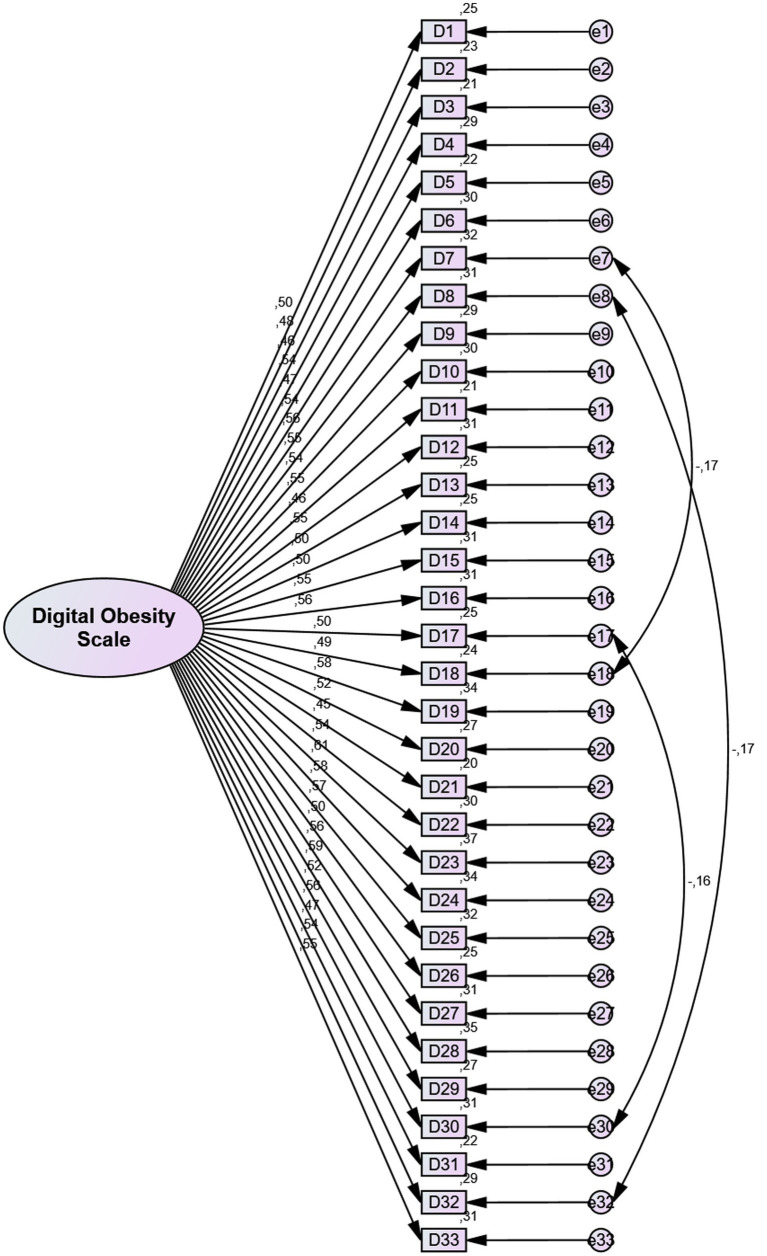
Confirmatory factor analysis (CFA) model for the Digital Obesity Scale showing the factor structure and standardized factor loadings.

The Digital Obesity Scale was developed by Koçoğlu et al. (31) in 2022 to evaluate individuals’ levels of digital obesity. The scale consists of a total of 33 items and five sub-dimensions: the ego surf sub-dimension (items 1–14), the dependency sub-dimension (items 15–23), the accessibility sub-dimension (items 24–28), the loading content sub-dimension (items 29–31) and the reference sub-dimension (items 32, and 33). The scale is a 5-point Likert-type scale, scored from 1 (strongly disagree) to 5 (strongly agree). Total scores range from 33 to 165. A high score obtained from the scale indicates a high level of digital obesity, while a low score obtained from the scale indicates a low level of digital obesity. The Cronbach’s alpha coefficient of the original scale was reported as 0.93 (31). In the present study, Cronbach’s alpha value was found to be 0.93. Digital Obesity Scale fit indices were determined as χ^2^/df = 1.113, RMSEA = 0.017, CFI = 0.983, GFI = 0.926, AGFI = 0.915, IFI = 0.984, TLI = 0.982. The CFA results indicated that the model demonstrated an acceptable fit (51) (Figure 3).

**Figure 4:**
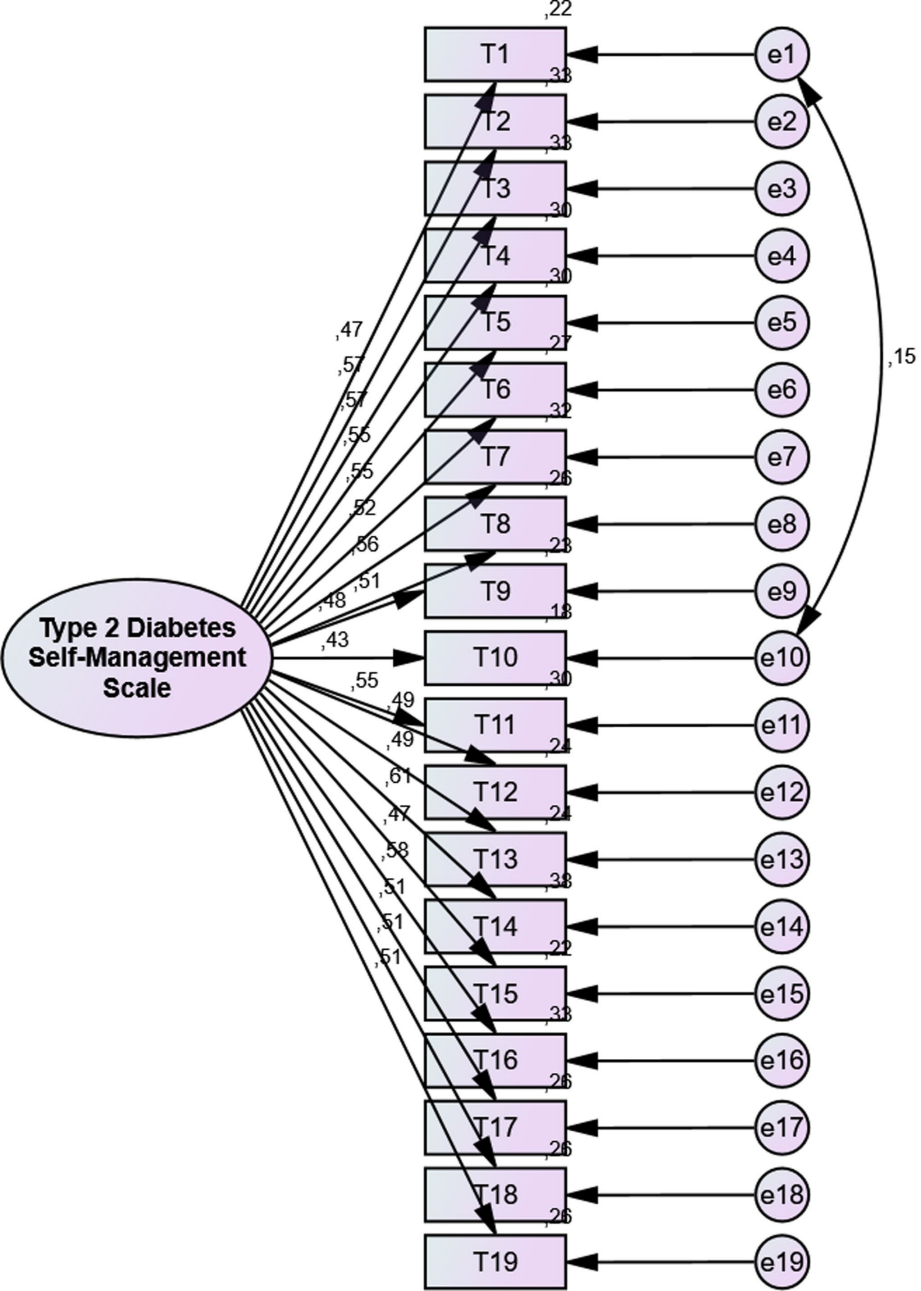
Confirmatory factor analysis (CFA) model for the type 2 diabetes self-management scale showing the factor structure and standardized factor loadings.

The Type 2 Diabetes Self-Management Scale was developed by Koç et al. (52) in 2024 to evaluate self-management behaviors of individuals with T2DM. The scale consists of a total of 19 items and three sub-dimensions: the healthy lifestyle behaviors sub-dimension (items 1–11), the use of health services sub-dimension (items 12–15) and the blood glucose management sub-dimension (items 16–19). The scale is a 5-point Likert-type scale, scored from 1 (never) to 5 (always). Total scores on the scale range from 19 to 95. A high score obtained from the scale indicates a good self-management, while a low score obtained from the scale indicates a poor self-management. The Cronbach’s alpha coefficient of the original scale was reported as 0.86 (52). In the present study, Cronbach’s alpha value was found to be 0.88. Type 2 Diabetes Self-Management Scale fit indices were determined as χ^2^/df = 1.100, RMSEA = 0.016, CFI = 0.991, GFI = 0.959, AGFI = 0.948, IFI = 0.991, TLI = 0.989. The CFA results supported the factorial validity of the scale (51) (Figure 4).

### 2.3 Statistical analysis

The analysis of the study data was performed by using SPSS version 23.0 software (IBM Corp., Armonk, NY, USA). Skewness and kurtosis values between −1.5 and +1.5 were considered indicative of a normal distribution (53). The data were normally distributed. Descriptive statistics are presented as mean (X̅), standard deviation (SD), minimum and maximum values for numerical variables, and frequency (n) and percentage (%) for categorical variables. To examine the construct validity of the measurement instruments, confirmatory factor analysis (CFA) was conducted using AMOS version 24.0. The model fit indices were evaluated according to commonly accepted criteria (51). Multiple regression analysis was performed to examine the associations of hedonic hunger and digital obesity with type 2 diabetes self-management. Prior to conducting the regression analysis, the underlying assumptions of the method were assessed. Linearity between dependent and independent variables was evaluated by visual inspection of scatter plots. The independence of residuals was assessed using the Durbin–Watson test, which indicated values within the acceptable range. Normality of residuals was examined using histograms and normal probability (Q–Q) plots, which indicated distributions consistent with normality. Homoscedasticity was assessed by examining residuals plotted against predicted values. Multicollinearity was evaluated using variance inflation factor (VIF) and tolerance values, all of which were within acceptable limits. Potential outliers were examined using Cook’s distance and Mahalanobis distance, and no influential observations were identified. The mediating role of digital obesity in the relationship between hedonic hunger and type 2 diabetes self-management was examined using Hayes’ PROCESS macro (Model 4) and the Bootstrap method (54). The statistical significance level was set at *p* < 0.05.

## 3 Results

### 3.1 General characteristics, body mass index (BMI) classification, and scale scores of the participants

It was determined that 51.5% of the participants were female, 84.5% were married, 36% were high school graduates, 64.0% were workers, 26.0% were current smoker, and 88.5% were non-drinker. While 74.5% were overweight or obese, 25.5% had normal body weight. The mean age of participants was 48.05 ± 6.60 years, the mean duration of diabetes was 7.68 ± 2.44 years, and the mean BMI was 27.47 ± 3.59 kg/m2. The mean scores for the Power of Food Scale, Digital Obesity Scale, and Type 2 Diabetes Self-Management Scale were 3.02 ± 0.79, 98.61 ± 25.42, and 57.59 ± 15.03, respectively (Table 1).

**Table 1:** General characteristics of the participants (*n* = 400).

Characters	*n*	%
Gender		
Female	206	51.5
Male	194	48.5
Marital status		
Married	338	84.5
Single	28	7.0
Widow/divorced	34	8.5
Educational level		
Secondary school	92	23.0
High school	144	36.0
Bachelor degree	126	31.5
Postgraduate degree	38	9.5
Working status		
Working	256	64.0
Nonworking	144	36.0
Smoking status		
Current smoker	104	26.0
Nonsmoker	174	43.5
Past smoker	122	30.5
Alcohol consumption		
Current drinker	30	7.5
Nondrinker	354	88.5
Past drinker	16	4.0
BMI classification		
Normal weight	102	25.5
Overweight	174	43.5
Obese	124	31.0
	X̅±SD (Min–Max)	
Age (year)	48.05 ± 6.60 (24.00–59.00)	
Duration of diabetes	7.68 ± 2.44 (2.00–16.00)	
BMI (kg/m^2^)	27.47 ± 3.59 (22.50–35.30)	
Power of Food Scale total score	3.02 ± 0.79 (1.23–5.00)	
Digital Obesity Scale total score	98.61 ± 25.42 (40.00–165.00)	
Type 2 Diabetes Self-Management Scale total score	57.59 ± 15.03 (23.00–90.00)	

BMI, body mass index, X̅±SD, Means ± standard deviation.

### 3.2 Prediction of type 2 diabetes self-management by hedonic hunger and digital obesity

Multiple linear regression analysis was conducted to examine the associations of hedonic hunger and digital obesity with type 2 diabetes self-management. The model was statistically significant [*F* (2, 397) = 579.65, *p* < 0.001]. It was found that the independent variables together explained 74.4% of the variance in type 2 diabetes self-management (adjusted R^2^ = 0.744).

According to the regression coefficients, hedonic hunger was significantly and negatively associated with type 2 diabetes self-management (*β* = −0.371, *p* < 0.001), and digital obesity was also significantly and negatively associated with type 2 diabetes self-management (*β* = −0.523, *p* < 0.001), which means that higher levels of hedonic hunger and digital obesity are associated with lower type 2 diabetes self-management scores. Multicollinearity diagnostics indicated that tolerance values (0.257) and VIF values (3.884) were within acceptable limits, suggesting no evidence of multicollinearity in the model (Table 2).

**Table 2:** Multiple linear regression analysis examining the association of hedonic hunger and digital obesity with type 2 diabetes self-management.

Independent variables	B	SE B	95% CI	*β*	t	*p*-value	Collinearity Statistics	
							Tolerance	VIF
Constant	109.484	1.572	[106.395, 112.574]		69.666	<0.001		
Hedonic hunger	−7.085	0.954	[−8.961, −5.208]	−0.371	−7.423	<0.001	0.257	3.884
Digital obesity	−0.309	0.030	[−0.367, −0.251]	−0.523	−10.464	<0.001	0.257	3.884

Model summary: R = 0.863, R^2^ = 0.745, Adjusted R^2^ = 0.744, F (2, 397) = 579.649, *p* < 0.001.

B = unstandardized regression coefficient; SE B = Standard error of B; β = standardized regression coefficient; CI = confidence interval; dependent variable: type 2 diabetes self-management.

### 3.3 Mediating role of digital obesity in the association between hedonic hunger and type 2 diabetes self-management

**Figure 5:**
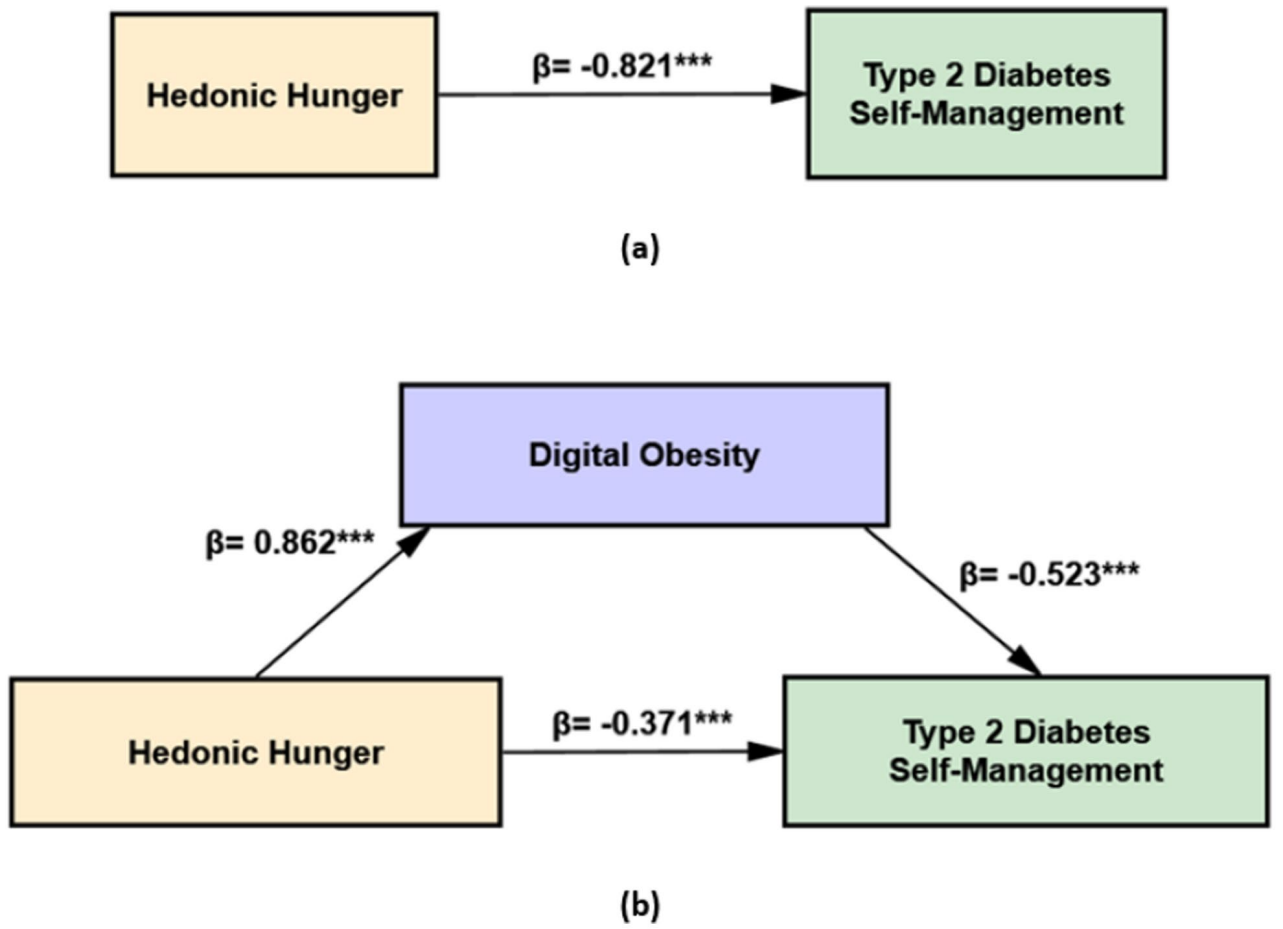
Mediation analysis showing the role of digital obesity in the association between hedonic hunger and type 2 diabetes self-management: **(a)** total effect model and **(b)** mediation model including digital obesity, showing the direct and indirect effects.

Mediation analysis was conducted using Hayes’ PROCESS macro (Model 4). The total effect of hedonic hunger on type 2 diabetes self-management (path c) was negative and statistically significant (B = −15.691, SE = 0.546, *p* < 0.001, 95% CI [−16.765, −14.617]) (Figure. 5a). Hedonic hunger was positively associated with digital obesity (path a; B = 27.850, SE = 0.822, *p* < 0.001, 95% CI [26.234, 29.466]). Digital obesity was negatively associated with type 2 diabetes self-management (path b; B = −0.309, SE = 0.030, *p* < 0.001, 95% CI [−0.367, −0.251]). The direct effect of hedonic hunger on type 2 diabetes self-management (path c′) was negative and statistically significant (B = −7.085, SE = 0.954, *p* < 0.001, 95% CI [−8.961, −5.208]). The indirect effect of hedonic hunger on type 2 diabetes self-management via digital obesity was also negative and statistically significant (B = −8.606, Boot SE = 0.874, 95% CI [−10.334, −6.903]). Taken together, these findings indicate that digital obesity partially mediates the association between hedonic hunger and type 2 diabetes self-management (Table 3). Standardised path coefficients for the mediation model are presented in Figure 5b.

**Table 3:** Mediation analysis using PROCESS Model 4 examining the mediating role of digital obesity in the association between hedonic hunger and type 2 diabetes self-management.

Model	B	SE B	95% CI	β	t	*p*-value
Hedonic hunger → Digital obesity (Path a)	27.850	0.822	[26.234, 29.466]	0.862	33.882	<0.001
Digital obesity → Type 2 diabetes self-management (Path b)	−0.309	0.030	[−0.367, −0.251]	−0.523	−10.464	<0.001
Hedonic Hunger → Type 2 diabetes self-management (direct effect: Path c^’^)	−7.085	0.954	[−8.961, −5.208]	−0.371	−7.423	<0.001
Hedonic Hunger → Type 2 diabetes self-management (total effect: Path c)	−15.691	0.546	[−16.765, −14.617]	−0.821	−28.722	<0.001

Model	B	BootSE (for B)	Bootstrap 95% CI (for B)	β	BootSE (for β)	Bootstrap 95% CI (for *β*)
Hedonic Hunger → Type 2 diabetes self-management (indirect effect: Path a*Path b)	−8.606	0.874	[−10.334, −6.903]	−0.450	0.044	[−0.537, −0.365]

B = unstandardized regression coefficient; SE B = Standard error of B; β = standardized regression coefficient; CI = confidence interval; indirect effects were estimated using bootstrapping.

Based on the results of the analyses, H1, H2, and H3 were supported. Furthermore, the mediation analysis confirmed H4, demonstrating that digital obesity mediates the association between hedonic hunger and type 2 diabetes self-management.

## 4 Discussion

The current study identified a partial mediating role of digital obesity in the association between hedonic hunger and type 2 diabetes self-management. Our findings indicated that higher hedonic hunger scores were negatively associated with type 2 diabetes self-management (*p* < 0.001). These findings are consistent with previous studies reporting that hedonic hunger and reward-driven eating behaviors are associated with poorer glycemic outcomes and difficulties in dietary adherence among individuals with T2DM (3, 12, 55). More specifically, Cheung et al. reported that hedonic hunger was associated with poorer glycaemic control among adults with T2DM, while Nicolau et al. showed that reward-related dysfunctional eating patterns were linked to worse metabolic control and diabetes-related complications (3, 12). Similarly, Petroni et al. reported that dysfunctional eating behaviors were common among individuals with T2DM and were associated with adverse clinical and psychosocial characteristics (55). From a behavioral perspective, hedonic hunger may undermine diabetes self-management by promoting preference for highly palatable, energy-dense foods and reducing adherence to dietary recommendations. Therefore, the negative association observed in the present study supports the view that reward-driven eating tendencies represent an important barrier to effective type 2 diabetes self-management.

In the present study, a significant positive association was observed between hedonic hunger and digital obesity. Higher levels of hedonic hunger were associated with higher levels of digital obesity, suggesting that individuals with stronger reward-driven eating tendencies may be more vulnerable to digital environments characterised by prolonged screen exposure and food-related cues. This finding is consistent with previous studies indicating that increased social media use and digital food-related content are associated with higher levels of hedonic hunger, unhealthy snacking behaviors, and greater consumption of energy-dense foods (37, 38, 41, 46). For example, Dumlu Bilgin et al. reported that social media usage was associated with both eating behavior and hedonic hunger, while Ryu et al. demonstrated that smartphone usage patterns were linked to dietary risk factors and unhealthy eating habits (37, 38). From this perspective, digital environments may amplify reward-driven eating tendencies by increasing exposure to highly palatable food cues and facilitating frequent food-related engagement. Therefore, the positive association observed in the present study suggests that hedonic hunger and digital lifestyle behaviors may interact in a way that reinforces unhealthy eating patterns, which may ultimately pose challenges for effective type 2 diabetes self-management.

Previous research has shown that prolonged screen time is associated with lower physical activity, increased sedentary behavior, and less healthy dietary patterns (39, 40, 56, 57). Physical inactivity has likewise been associated with reduced insulin sensitivity and poorer glycaemic control, which are critical components of effective type 2 diabetes self-management (58). In addition, exposure to digital food advertising and online food-related content has been linked to increased consumption of energy-dense, nutrient-poor foods, which may hinder adherence to dietary recommendations among individuals with type 2 diabetes (35, 36). Such patterns may reduce compliance with dietary and lifestyle behaviors that are central to effective diabetes self-management. Taken together, these findings suggest that digital lifestyle factors may represent a behavioral context that negatively influences type 2 diabetes self-management by simultaneously reducing physical activity opportunities and increasing exposure to unhealthy food cues. Accordingly, consistent with the existing literature, the present study demonstrated that higher levels of digital obesity were significantly associated with lower levels of type 2 diabetes self-management (*p* < 0.001).

Overall, the principal findings of this study indicate that higher levels of hedonic hunger are associated with poorer type 2 diabetes self-management, and that this association is partly explained by digital lifestyle habits, including excessive screen use, sedentary behavior, and exposure to online food-related stimuli. Hedonic hunger, characterised by heightened food cravings among individuals with T2DM, has been associated with lower adherence to healthy dietary patterns and poorer glycemic control (59). Our findings further suggest that digital lifestyle habits may intensify the association between hedonic hunger and suboptimal type 2 diabetes self-management, consistent with the observed partial mediating role of digital obesity. In contemporary digital environments, frequent exposure to food-related content, advertising, and online food delivery platforms has been linked to increased consumption of energy-dense foods, particularly among individuals with elevated hedonic hunger (22). In addition, sedentary behaviors commonly associated with digital obesity have been associated with reduced insulin sensitivity and challenges in achieving glycemic control (58). Taken together, these findings indicate that the co-occurrence of hedonic hunger and digital obesity represents an important risk context for type 2 diabetes self-management rather than independent influences. From a public health perspective, the results underscore the importance of addressing both psychological eating drives and digital lifestyle factors when considering diabetes self-management. Interventions that incorporate strategies to manage hedonic eating tendencies and promote healthier digital behaviors may represent valuable components of comprehensive type 2 diabetes self-management programmes. Individuals with elevated hedonic hunger may be particularly vulnerable to digital environments that promote exposure to highly palatable foods and sedentary behaviors. Therefore, diabetes education and behavioral interventions may benefit from incorporating strategies that help individuals regulate reward-driven eating tendencies while also promoting healthier digital habits, such as reducing excessive screen time and increasing engagement in physical activity.

This study has several limitations. First, the cross-sectional design precludes causal inference. Second, the use of online self-reported data may have introduced recall bias, social desirability bias, and self-report measurement error. In particular, self-reported anthropometric data such as body weight and height may be subject to reporting inaccuracies, which could affect BMI estimation. Third, the use of a snowball sampling strategy and online recruitment may have introduced selection bias, potentially limiting the representativeness of the sample. Finally, the sample consisted of individuals with T2DM residing in Turkey, which may limit the generalizability of the findings to other populations. In addition, the present study did not examine potential differences across demographic or clinical subgroups. Future research may explore whether the relationships among hedonic hunger, digital obesity, and type 2 diabetes self-management differ across subgroups such as age, gender, BMI status, or duration of diabetes. Examining potential heterogeneity across patient characteristics may provide further insights into how behavioral and lifestyle factors influence diabetes self-management in different populations. Furthermore, longitudinal and experimental studies are needed to better understand the temporal and causal pathways linking hedonic hunger, digital obesity, and type 2 diabetes self-management.

## 5 Conclusion

This study highlights that digital obesity mediates the association between hedonic hunger and type 2 diabetes self-management. By examining both the individual and combined associations of hedonic hunger and digital obesity with diabetes self-management among individuals with T2DM, the present findings address an important gap in the existing literature and contribute to a more comprehensive understanding of behavioral and lifestyle factors relevant to diabetes management. From a broader perspective, these findings underscore the potential relevance of integrating psychological eating tendencies and digital lifestyle factors within multidisciplinary approaches to diabetes self-management.

## Data Availability

The raw data supporting the conclusions of this article will be made available by the authors, without undue reservation.
